# Whole-genome sequencing of Streptococcus uberis isolated from cows with mastitis in Thuringia

**DOI:** 10.1099/jmm.0.001887

**Published:** 2024-10-16

**Authors:** Christine Thomas, Jörg Linde, Hosny El-Adawy, Nadine Wedlich, Katja Hruschka, Esra Einax, Karsten Donat, Christian Berens, Herbert Tomaso

**Affiliations:** 1 Institute of Bacterial Infections and Zoonoses, Federal Research Institute for Animal Health, Friedrich-Loeffler-Institute, Jena, Germany; 2 RNA Bioinformatics and High-Throughput Analysis, Friedrich Schiller University Jena, Jena, Germany; 3 Faculty of Veterinary Medicine, Kafrelsheikh University, Kafr El-Sheikh, Egypt; 4 Tiergesundheitsdienst der Thueringer Tierseuchenkasse, Jena, Germany; 5 Institute of Molecular Pathogenesis, Federal Research Institute for Animal Health, Friedrich-Loeffler-Institute, Jena, Germany

**Keywords:** AMR, bovine mastitis, MLST, plasmids, *Streptococcus uberis*

## Abstract

**Introduction.** *Streptococcus uberis* is a common cause of mastitis in cattle, leading to significant economic losses. The widespread use of antimicrobials has contributed to the emergence of resistance, which poses a severe challenge in controlling *S. uberis* infection.

**Aim.** The objective of this study was to gain insights into the antimicrobial resistance (AMR) and epidemiological typing of *S. uberis* isolated from milk collected from bovine mastitis on dairy farms in Thuringia.

**Methodology.** In this study, 84 *S. uberis* isolates were obtained from cattle with clinical mastitis in Thuringia, their phenotypic and genotypic AMR were analyzed and their phylogenetic relationship was explored using whole-genome sequencing.

**Results.** Genetically heterogeneous strains were found on the farms, but clusters of highly similar strains also circulated within the same farms. All isolates were sensitive to ampicillin, penicillin, ceftiofur, and vancomycin. However, 42.9%, 42.9%, 22.6%, 19.0%, and 13.0% were resistant to tetracycline, doxycycline, clindamycin, pirlimycin, and erythromycin, respectively. Thirty-nine strains were phenotypically resistant to two or more tested antibiotics. We identified a plasmid associated with macrolide and lincosamide resistance in 12% of the strains.

**Conclusion.** The emergence of *S. uberis* strains resistant to multiple antibiotics highlights the importance of *S. uberis* surveillance and the prudent use of antimicrobials.

**Impact Statement**

This study on *Streptococcus uberis* in cattle with mastitis in Thuringia, Germany, revealed a high degree of genetic diversity of *S. uberis* on farms. No strains were phenotypically resistant to beta-lactam antibiotics, but decreased susceptibility was found for six strains. The findings have implications for farm management practices, antibiotic stewardship and the ongoing challenge of mitigating antimicrobial resistance in the context of bovine mastitis.

## Data Summary

The data presented in the study have been deposited in the European Nucleotide Archive (ENA) repository, project accession number PRJEB67942. Data are accessible via https://www.ebi.ac.uk/ena/browser/view/PRJEB67942.

Reference genome *Streptococcus uberis* NCTC4672, accession no. GCF_900460135.1 (NCBI, https://www.ncbi.nlm.nih.gov/datasets/genome/GCF_900460135.1/) [1,2].

The authors confirm that all supporting data, code and protocols have been provided in the article or through supplementary data files.

## Introduction

Bovine mastitis is the most frequent disease in cattle and causes high economic losses [3]. The disease is defined as an inflammation of the udder and is associated with increased somatic cell count (SCC). Clinical mastitis usually presents with mild to severe swelling of the udder, redness and pain. Depending on the severity of the inflammation, the milk may contain flakes or blood, and the body temperature may be increased [4,6]. Subclinical mastitis is diagnosed based on elevated SCC and pathogen detection in the milk [7].

The treatment of clinical mastitis accounts for significant antimicrobial consumption in dairy herds [8,9]. Selective dry cow therapy for treating only selected cattle with intramammary infections is a common treatment strategy, as blanket dry cow therapy with antimicrobials used for preventive purposes has been banned in the EU since January 2022 (EU Regulation 2019/6) [10]. The selection of cows that need treatment can be based on approaches such as frequent bacteriological analysis and computer-automated data-driven algorithms [11].

In recent years, *Streptococcus uberis*, as a cause of mastitis, has become an increasing concern [12,15]. While these Gram-positive bacteria are typically considered to be environmental pathogens, *S. uberis* might occasionally be transmitted from cow to cow [16,23]. The bacterial population of *S. uberis* is often diverse, yet genetically highly similar strains have been identified [24].

In the context of One Health, the role of livestock as potential sources of antimicrobial-resistant bacteria or resistance-conferring genes is a known concern [25]. Extensive antimicrobial use has been linked to the development of antimicrobial resistance (AMR) across various microorganisms [26,28]. Although a study by the Federal Office of Consumer Protection and Food Safety indicates a reduction in antibiotic use in Germany [29], mastitis remains a primary reason for antimicrobial treatment on dairy farms [30].

*S. uberis* is not intrinsically resistant to penicillin, but decreased susceptibility has been reported in several regions (Canada [31], Europe [32,34], New Zealand [35], and Brazil [36]). Penicillin and other beta-lactam antibiotics are commonly employed in the local treatment of streptococcal infections [24,37]. Studies have also highlighted the occasional resistance of *S. uberis* to macrolides, lincosamides and tetracyclines [38,40].

Mobile genetic elements (MEG) such as plasmids are essential for rapid dissemination of antibiotic-resistance genes among bacteria [41].

This study aims to investigate the genomic profiles of *S. uberis* strains causing bovine mastitis in Thuringia, Germany, with a particular focus on their phenotypic and genotypic AMR profiles. Milk samples from cattle with bovine mastitis were collected consecutively at the Animal Health Services [Tiergesundheitsdienste (TGD)] in Thuringia in 2021 and 2022. Eighty-four *S. uberis* strains were isolated, and their phenotypic AMR profiles were determined. All strains were sequenced with short-read technology to assess the presence of genetic markers for AMR and to perform molecular typing. Furthermore, 24 strains representing the major clades were sequenced using long-read technology to characterize potential plasmids carrying AMR-associated genes.

## Methods

### Bacterial strains

Eighty-four *S. uberis* strains were isolated from milk samples of dairy cows with clinical mastitis (diagnosed during routine screening) during 2021 and 2022 from 40 dairy farms located in Thuringia, Germany, and kindly provided from the diagnostic laboratory of the Animal Health Services of Thuringia (TGD). Species identification of the 84 *S*. *uberis* strains was confirmed using matrix-assisted laser desorption ionization time-of-flight mass spectrometry (MALDI–TOF MS). All strains were stored at −80 °C until further investigations.

### Phenotypic antimicrobial resistance

The minimum inhibitoy concentration (MIC) of nine antimicrobial agents [ampicillin (AMP), ceftiofur (CET), clindamycin (CLI), doxycycline (DOX), erythromycin (ERY), penicillin (PEN), pirlimycin (PIR), tetracycline (TET) and vancomycin (VAN)] belonging to six classes were determined for all 84 *S*. *uberis* strains using the MICRONAUT broth dilution system (Merlin, Bornheim-Hersel, Germany). The specialized plates for mastitis (MASTITIS-S) and equine/livestock for Gram-positive bacteria (E/L for GP) were used for the antimicrobial susceptibility testing (AST). The antibiotics tested in this study were frequently used antimicrobial agents for the treatment of mastitis and for use in human medicine [34, 40,42,46]. Breakpoints were applied according to the Clinical & Laboratory Standards Institute (CLSI) Vet01S (third edition) standards [47]. For three antibiotics (clindamycin, penicillin and pirlimycin), species-specific breakpoints were selected [47]. If no animal or species-specific cutoffs were specified, the breakpoints for *Streptococcus* (*viridans* group) or the breakpoints based on human data were applied as recommended by CLSI [47].

### Whole-genome sequencing with Illumina and Oxford Nanopore Technologies

All 84 *S*. *uberis* strains were sequenced with an Illumina MiSeq (Illumina GmbH, Munich, Germany). A subset of 24 *S*. *uberis* strains representing the major clades and plasmid-content profiles were additionally sequenced with a MinION device [Oxford Nanopore Technologies (ONT), UK]. For Illumina sequencing, the DNA of all 84 *S*. *uberis* strains was extracted and purified using the QIAGEN Genomic-tip 20 G Kit (QIAGEN, Germany) and the Genomic DNA Buffer Set (QIAGEN, Germany). The concentration of the DNA was determined using the Qubit dsDNA BR assay kit (Invitrogen, USA). Sequencing libraries were created using the Nextera XT DNA Library Preparation Kit (Illumina Inc., USA). Paired-end sequencing with two cycles of 301 bps was performed on an Illumina MiSeq instrument according to the manufacturer’s instructions (Illumina Inc., USA).

Long-read sequencing was performed using a MinION device. The DNA of 24 *S*. *uberis* strains was extracted with the QIAGEN Genomic-tip 20 G Kit (QIAGEN, Germany) and the Genomic DNA Buffer Set (QIAGEN, Germany). Barcoding was done using the Ligation Sequencing Kit (SQK-LSK114) and the Native Barcoding Expansion Kit (SQK-NBD114.24). The sequencing was performed with an R10.4.1 flow cell (FLO-MIN114) according to the manufacturer’s instructions.

### Bioinformatic analysis

If no specific settings are mentioned, the tools utilized for the bioinformatic analyses were used in their respective default settings.

Illumina reads were assembled using the pipeline WGSBAC (v 2.2.3) [48]. Here, the raw paired-end reads served as input, the coverage was calculated and the quality of the short-read sequencing data was accessed with FastQC [49]. A coverage >30 was considered acceptable for further analyses. Quality and adapter trimming of Illumina reads and assembly were performed by Shovill v. 1.0.4 [50].

ONT reads were combined with the Illumina reads to reconstruct hybrid assemblies. Basecalling, trimming, assembly, and polishing of ONT data were performed with the Linux-based bioinformatic pipeline BONT (https://gitlab.com/ChristineThms/bacteriapipeline). Guppy v 6.5.7 with the dna_r10.4.1_450bps_hac.cfg model was applied for basecalling and demultiplexing. The quality was checked with NanoPlot v 1.41.3. Hybrid assemblies were built with unicycler [51] as proposed by Johnson *et al*. [52].

The polished hybrid assemblies (unicycler) and the short-read-only assemblies were further analysed with the WGSBAC pipeline. Assembly quality was assessed with QUAST v 5.2.0 [53]. Genome annotation was performed with Bakta v 1.8.2 [54], and the assemblies were checked for contamination with Kraken2 v 2.1.3 [55].

Multilocus sequence typing (MLST) was performed with the software mlst v2.23.0 [56] based on the database PubMLST *S. uberis* MLST scheme [57]. New sequence types (STs) were uploaded to PubMLST. To identify the core-genome SNP alignment (cgSNPs), Snippy v 4.6.0 [58] was applied to map the raw reads to the hybrid assembly of 22CS0234. The cgSNP alignment and RAxML v 8.2.12 [59] with the GTRGAMMA model and rapid bootstrapping were used to reconstruct the phylogenetic tree. The resulting best-scoring maximum likelihood (ML) tree was visualized with ggtree [60] and treeio [61] using 21CS0142 as root. Additionally, hierarchical clustering based on the distance matrix of cgSNPs, generated with snp-dists v 0.7.0 [58], was performed with threshold values of 200 cgSNPs. The cutoff value was selected based on the silhouette score [62], the Davies–Bouldin index [63] and the Adjusted Rand Index [64] (data not shown). AMRFinderPlus v 3.11.18 [65], ResFinder [66], and CARD [67] were used to detect genetic markers for AMR (genes and mutations). Genes coding for penicillin-binding proteins (PBP) PBP2b, PBP2a, PBP1b, PBP1a and PBP2x were detected with ABRicate v 1.0.1 [68] for all strains. Mutations within these genes were determined with snippy v 4.6.0 [58]. ABRicate v 1.0.1 [68] with the database PlasmidFinder [69] was used to detect plasmid replicons for both short-read and long-read data. Putative AMR plasmids were derived from hybrid assemblies and considered when the contig included a replicon (found in long-read and short-read data) and AMR genes. Additionally, Platon was applied to differentiate plasmid-borne and chromosomal contigs [70]. A mapping approach with minimap2 and the possible AMR plasmids as a reference were used to investigate the occurrence in only short-read sequenced strains. Visualization of plasmids was performed with PlasMapper 3.0 [71]. Annotation of plasmids was performed with Bakta v1.7.0 [54]. Additionally, plasmid coding sequences (CDS) were further analysed with BLASTp [72] and NCBI CDD [73,74] to refine functional annotation.

## Results

### Phenotypic antimicrobial susceptibility profiles of *S. uberis* in Thuringia

AST of all 84 strains was performed for ampicillin, ceftiofur, clindamycin, doxycycline, erythromycin, penicillin, pirlimycin, tetracycline and vancomycin (Table 1). All isolates were sensitive to ampicillin, penicillin, ceftiofur and vancomycin. Phenotypic resistance against tetracycline, doxycycline, clindamycin, pirlimycin and erythromycin was observed (Table 1). A table with phenotypic AMR profiles of all strains is provided in the supplementary data (Supplementary material S1, available in the online Supplementary Material).

**Table 1.** Results of the phenotypic antimicrobial resistance of 84 *S. uberis* strains.

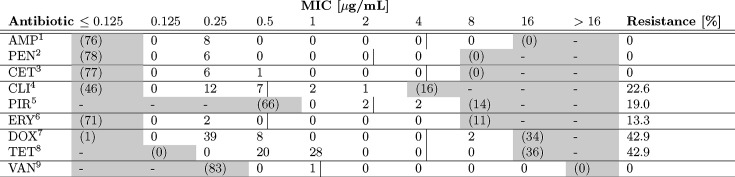 ­

1Ampicillin, 2Penicillin G, 3Ceftiofur, 4Clindamycin, 5Pirlimycin, 6Erythromycin, 7Doxycycline, 8Tetracycline, 9Vancomycin.

White fields indicate the test range for each antimicrobial substance; () indicates the number of strains with growth outside of the test range, corresponding to ≤ or > compared to the last measured antibiotic concentration. The black lines show the breakpoints.

**Table 2.** Comparison of AMR phenotype and genotype analyses for 84 *S. uberis* strains. Genetic marker determination for AMR was performed with ABRicate based on the AMRFinder, ResFinder and CARD databases.

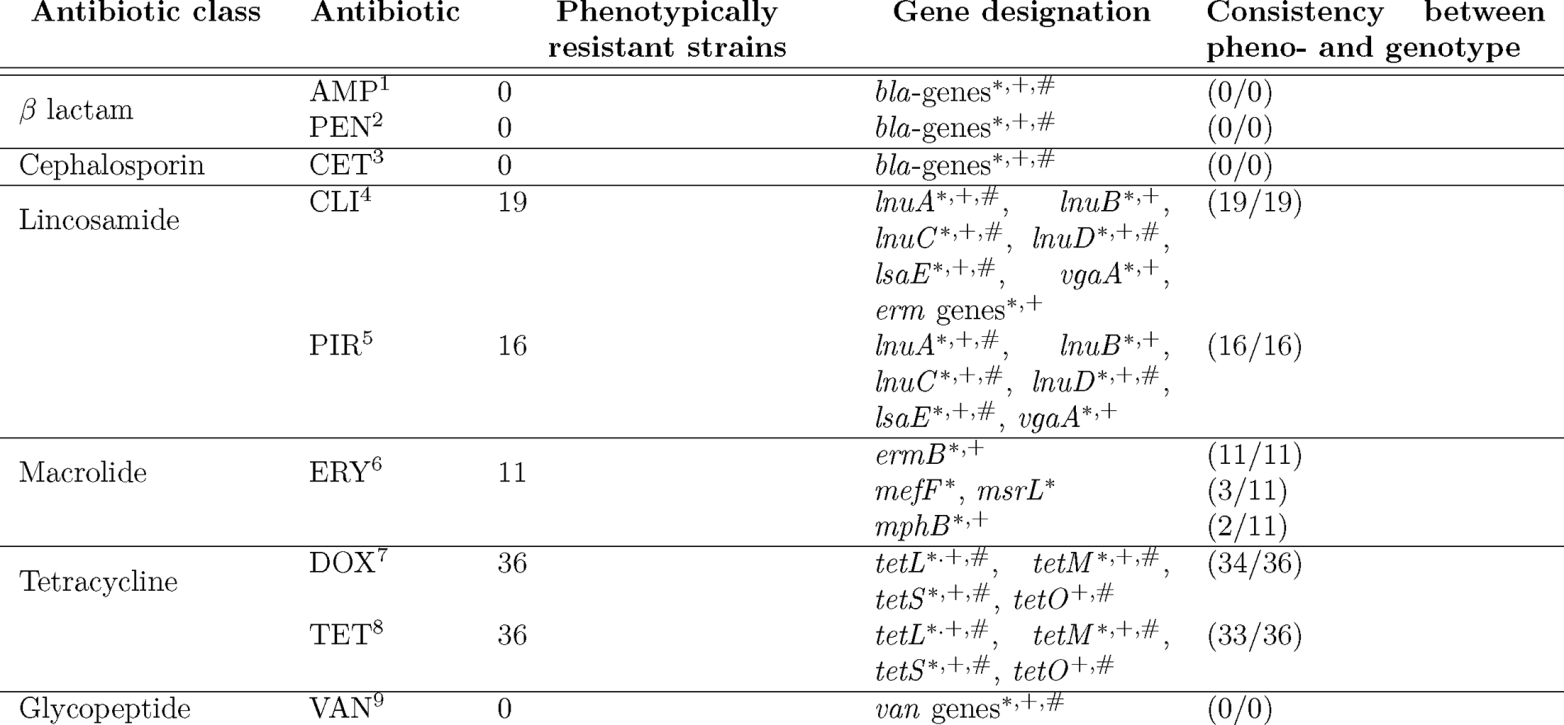 ­

1Ampicillin, 2Penicillin G, 3Ceftiofur, 4Clindamycin, 5Pirlimycin, 6Erythromycin, 7Doxycycline, 8Tetracycline, 9Vancomycin Genes marked are part of corresponding database: *AMRFinder, +ResFinder, #CARD.

### Genomic characteristics of *S. uberis* in Thuringia

Whole-genome sequencing (WGS) was performed for all 84 isolates of *S. uberis* (Supplementary material S2). Illumina sequencing yielded an average of 1 113 926 reads per isolate. The theoretical average coverage achieved for the reference genome GCF_900460135.1 is 268 (mean), and approximately 84.4% (mean) of the reads reach a quality score of Q30 on average. The assembled genomes consisted on average of 23 contigs (7 to 314) with a GC content of 36–37%. The length of Illumina-based assemblies ranged from 1 785 478 bps to 2 114 496 bps. In comparison, the reference genome comprised three contigs, had a GC content of 36.7%, and a total length of 1 966 403 bps.

For the 24 isolates sequenced with ONT, the average number of reads per isolate was 371 760 and had a mean read length of 3341 bps. The average percentage of Q15 quality of bases was 52.7%. This resulted in a theoretical coverage ranging from 32 to 2033 (mean 640) when considering the reference genome GCF_900460135.1. The resulting ONT assemblies consisted of 1 to 39 contigs, and the average GC content was 36–37%. The genome size of the various ONT assemblies ranged from 1 834 426 to 2 130 167 bps.

### Phylogeography of *S. uberis* in Thuringia

*In silico* MLST was conducted for all 84 isolates, revealing a total of 58 different STs (Supplementary material S3). Out of 58 STs, 43 were newly identified STs and were uploaded to PubMLST. The most prevalent ST identified in this study was ST 1984 (seven strains, Supplementary material S3). At farm Q, seven different STs were isolated over a 10-month time period.

**Fig. 1.**
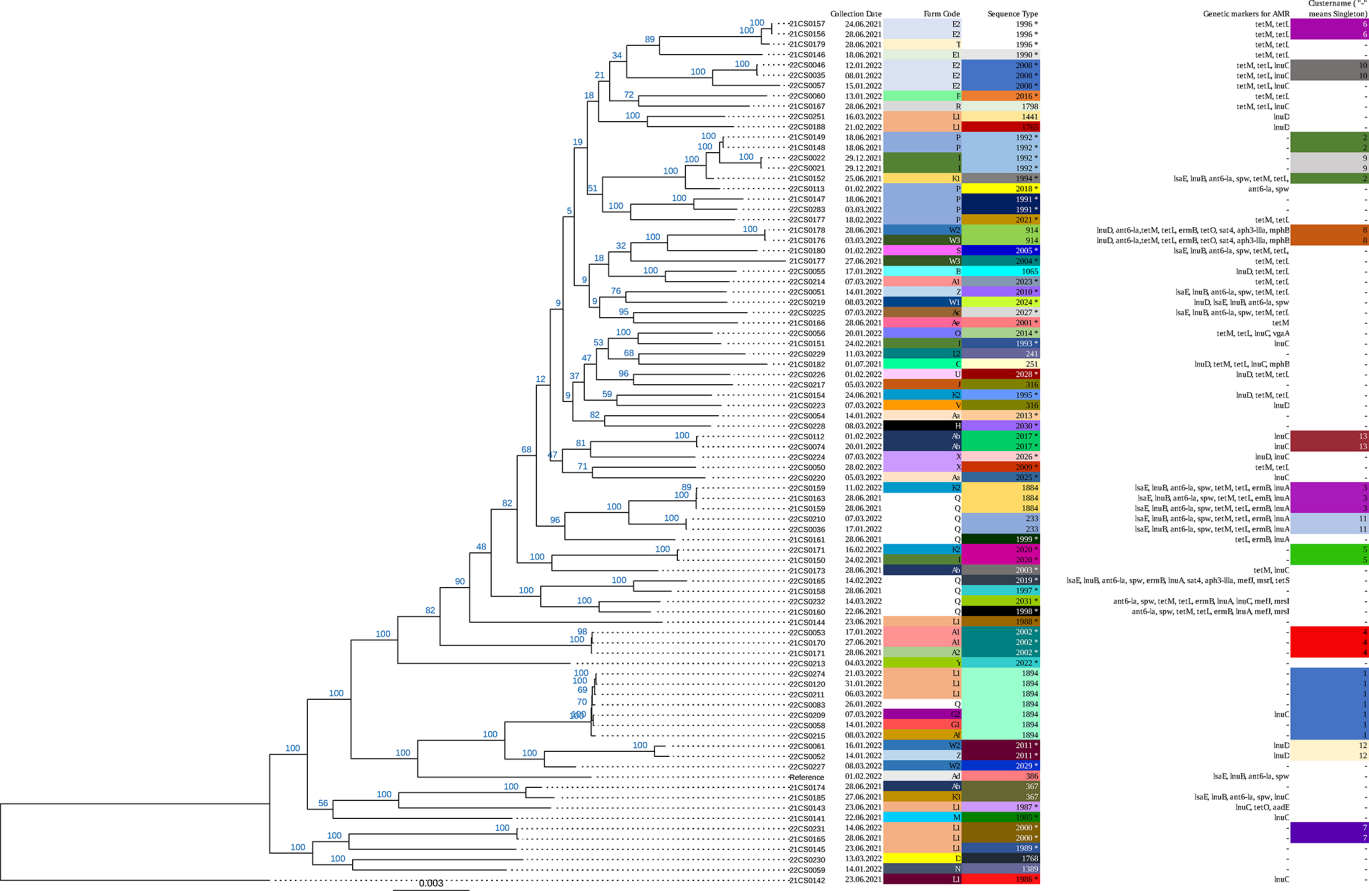
cgSNP-based phylogeny of 84 *S. uberis* strains. The fields indicate the date of collection, farm code, sequence type, genetic markers for antimicrobial resistance and the cluster designation. A * behind the sequence type marks a newly identified type.

A high-resolution phylogeny was constructed using a cgSNP alignment consisting of 34 977 genomic positions (Fig. 1). Pairwise cgSNP distances between two isolates ranged from 0 to 8966 cgSNPs (4782 cgSNPs on average). STs correlated with the major branches of the phylogenetic tree constructed based on the cgSNP alignment.

**Fig. 2.**
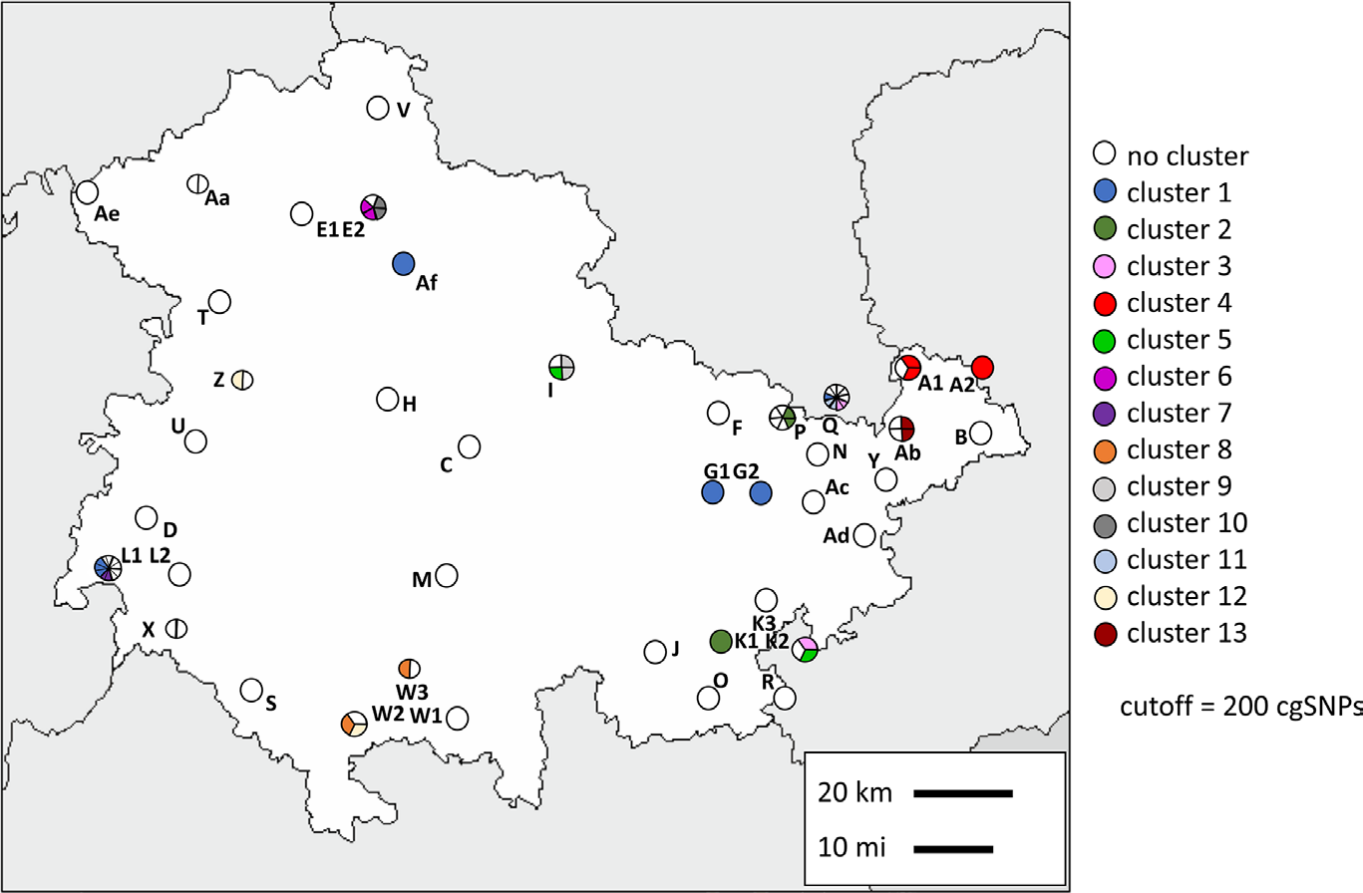
Map with strains collected per farm with their corresponding clusters. Farms with the same letter and a number in the name were located in the same postal code area. Each circle arc represents one strain collected from the corresponding farm. Clusters were sorted according to their size with cluster 1 being the largest cluster.

Pairwise SNP distances were employed for hierarchical clustering. Using a threshold of 200 cgSNPs, 13 clusters were detected (Supplementary material S4) consisting of 34 strains, whereas 50 strains remained singletons. Isolates from the same farm were usually genetically heterogeneous (Fig. 2). For example, on Farm L1, eleven strains were isolated between June 2021 and July 2022 and differed on average in 6370 cgSNPs.

However, genetically closely related strains persisted on a single farm and were also transmitted between farms (Fig. 2). The strains 21CS0171 and 22CS0053 in cluster 4 were isolated at farm A1 within a time span of six months and differed in 16 cgSNPs. Cluster 5 contained two strains (21CS0150 and 22CS0171) differing by 45 cgSNPs, which were collected from farms I and K2 that are 60 km distant from each other over a time span of six months. The largest cluster (cluster 1) comprised 7 of 84 strains from five farms distributed throughout Thuringia (Fig. 2). Cluster 2 comprised three strains collected from two farms. Here, 21CS0148 and 21CS0149 differed in nine cgSNPs. The remaining clusters consisted of two to three strains. Eight clusters (clusters 4, 6, 7, 8, 9, 10, 11 and 13) were confined to geographical regions. Clusters 6, 7, 9, 10, 11 and 13 were found only on a single farm, while clusters 4 and 8 were present on two farms (Fig. 1).

### Genetic markers for antimicrobial resistance of *S. uberis* in Thuringia

All strains were examined for the presence of AMR genes associated with resistance to beta-lactams, lincosamide, streptogramin, aminoglycosides, tetracycline, streptothricin and macrolides, using ABRicate (Tables1  2, Supplementary material S5).

No strain was resistant to penicillin, while six strains displayed decreased susceptibility (21CS0160, 21CS0166, 22CS0054, 22CS0159, 22CS0229, and 22CS0232), characterized by MIC values between 0.25 and 0.5. In all 84 strains, the PBP coding genes *pbp2a, pbp2b, pbp1b, pbp1a* and *pbp2x* were detected. Point mutations and insertions/deletions within the PBP-coding genes were found in all 84 strains at various positions compared to the reference strain (Supplementary material S6). For 57 positions in these genes, all 84 strains included the same substitution, and for eight positions, all 84 strains had insertions/deletions. Two strains with decreased susceptibility to penicillin (21CS0160 and 22CS0229) carried unique mutations, leading to substitutions in *pbp2a* (E_279_K) and in *pbp1b* (T_542_A), respectively. No other mutations within the PBP-coding genes were identified exclusively in those strains with decreased susceptibility. No known genetic marker conferring resistance to ampicillin and ceftiofur was detected in the assemblies.

Sixteen strains (19.0%) were phenotypically resistant against pirlimycin. The same set of strains also tested resistant against clindamycin. Additionally, strains 21CS0154, 21CS0158 and 22CS0055 also showed resistance to clindamycin. All resistant strains had at least one hit for a genetic marker for lincosamide resistance (*lnuD*, *lnuC*, *lnuA*, *lsaE*, *vgaA* and *lnuB*).

Most strains resistant to pirlimycin were also resistant to erythromycin, except for strains 21CS0150, 21CS0180, 21CS0185, 22CS0051 and 22CS0225. In all strains resistant to erythromycin (13.0%), the genetic marker *ermB* was identified. *ErmB* was only detected in one other strain, 22CS0159, which has a susceptible phenotype. The genes *mefJ, msrI* and *mphB* were detected in strains 21CS0176, 21CS0178 and 21CS0182 (Supplementary material S6).

Resistance to doxycycline and tetracycline was found in 42.9% of the strains. The same strains demonstrated resistance against both doxycycline and tetracycline, except for 21CS0147 (resistant to tetracycline only) and 21CS0163 (resistant to doxycycline only). Except for three strains (21CS0147, 21CS0150 and 22CS0219), all resistant strains harboured one or multiple genetic markers for tetracycline resistance (*tetM, tetL, tetS* and *tetO*). Two strains (21CS0152 and 22CS0159) carry one or multiple genetic markers for tetracycline resistance without displaying phenotypic resistance. Strains 21CS0147, 21CS0150 and 22CS0219 tested phenotypically resistant to tetracycline, but no genetic marker for AMR was detected.

Genetic markers coding for resistance against multiple antibiotic classes were detected (Supplementary material S5). Twenty-nine strains with no genetic marker for resistance detected were susceptible to all tested antibiotics. Twenty strains carried genetic markers conferring resistance to at least three antibiotic classes. The largest number of genetic factors were contained within the assembly of strain 22CS0165, carrying eleven markers. While two of these factors (*lsaE* and *ermB*) were multi-AMR genes, three different genetic factors (*mefJ*, *msrI* and *ermB*) potentially induced resistance to macrolides. In sum, 22CS0165 contained genetic factors conferring resistance to lincosamides, streptogramin, aminoglycosides, tetracyclines, streptothricin and macrolides. The strains 21CS0176 and 21CS0178 carried nine different genetic markers coding for resistance to the same set of antibiotic classes. These three strains were phenotypically resistant to lincosamides, macrolides and tetracyclines. AST was not performed for streptogramin, aminoglycosides and streptothricin.

### Plasmids present in *S. uberis* in Thuringia

Based on Illumina data, plasmid replicons were detected in 39 *S. uberis* with PlasmidFinder (Supplementary material S7). The short-read results were confirmed within 24 hybrid assemblies (Supplementary material S7). 22CS0159 might have lost the plasmid during re-cultivation for ONT sequencing and phenotypic AMR testing.

**Fig. 3.**
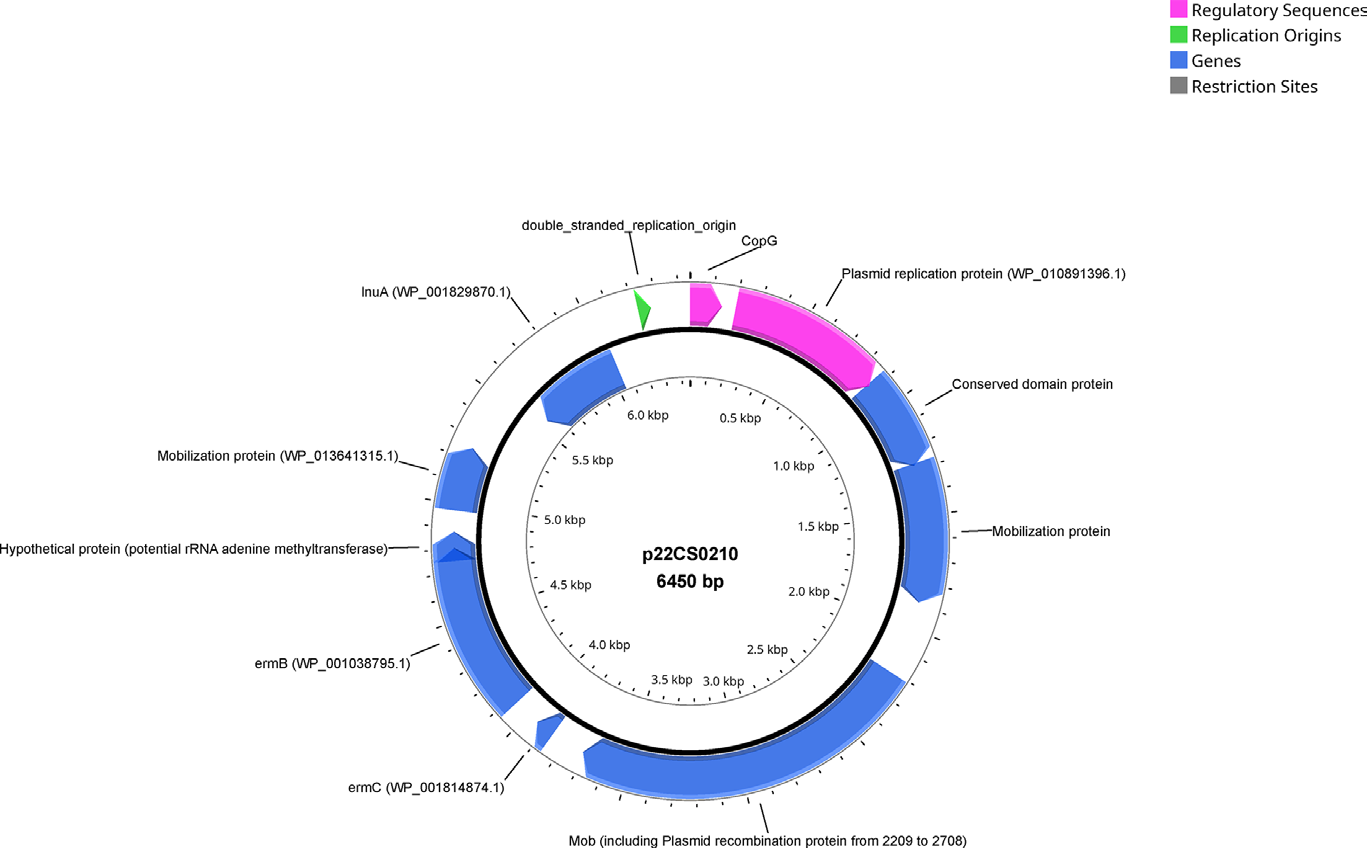
Plasmid p22CS0210 carrying two AMR genes found in 10 out of 84 *S. uberis* strains. Visualization of plasmid p22CS0210 with PlasMapper 3.0.

Seven potential plasmids were identified upon investigating the long-read assemblies (considering contigs with a replicon present within the hybrid assemblies). Notably, only one of these plasmids carried genes linked to antibiotic resistance (Fig. 3). To assess whether this plasmid could be detected in the Illumina sequencing data, a mapping approach of reads to the desired plasmid was performed for all strains. The plasmid was found in nine strains that contained the two AMR genes *lnuA* and *ermB* and repUS55_1_repB.pSSU1 on the same contig, while strain 22CS0165 carried the three markers on different contigs. Additionally, Platon results indicated that these contigs are plasmid-borne (Supplementary material S7). These strains all exhibited phenotypic resistance to pirlimycin, clindamycin and erythromycin. While *ermB* was additionally detected within the chromosome of two strains (21CS0176 and 21CS0178), *lnuA* was exclusively located on the plasmid p22CS0210. Strain 22CS0159 lost phenotypic resistance and the replicon during re-cultivation for ONT sequencing.

This hypothetical plasmid, designated as p22CS0210, had a length of 6450 bps and contained ten CDS, nine with predicted functional annotation and one coding for a hypothetical protein. The replication repressor *copG* was present on the plasmid, as well as the plasmid replicon repUS55_1_repB.pSSU1. P22CS0210 codes for three mobilization proteins, one of them including a plasmid-associated recombinase. Genes coding for the *ermC* rRNA methylase leader protein and the *ermB*-methyltransferase were also identified on the positive strand, along with a gene coding for a hypothetical protein. On the negative strand, the *lnuA* lincosamide nucleotidyltransferase gene was detected.

**Fig. 4.**
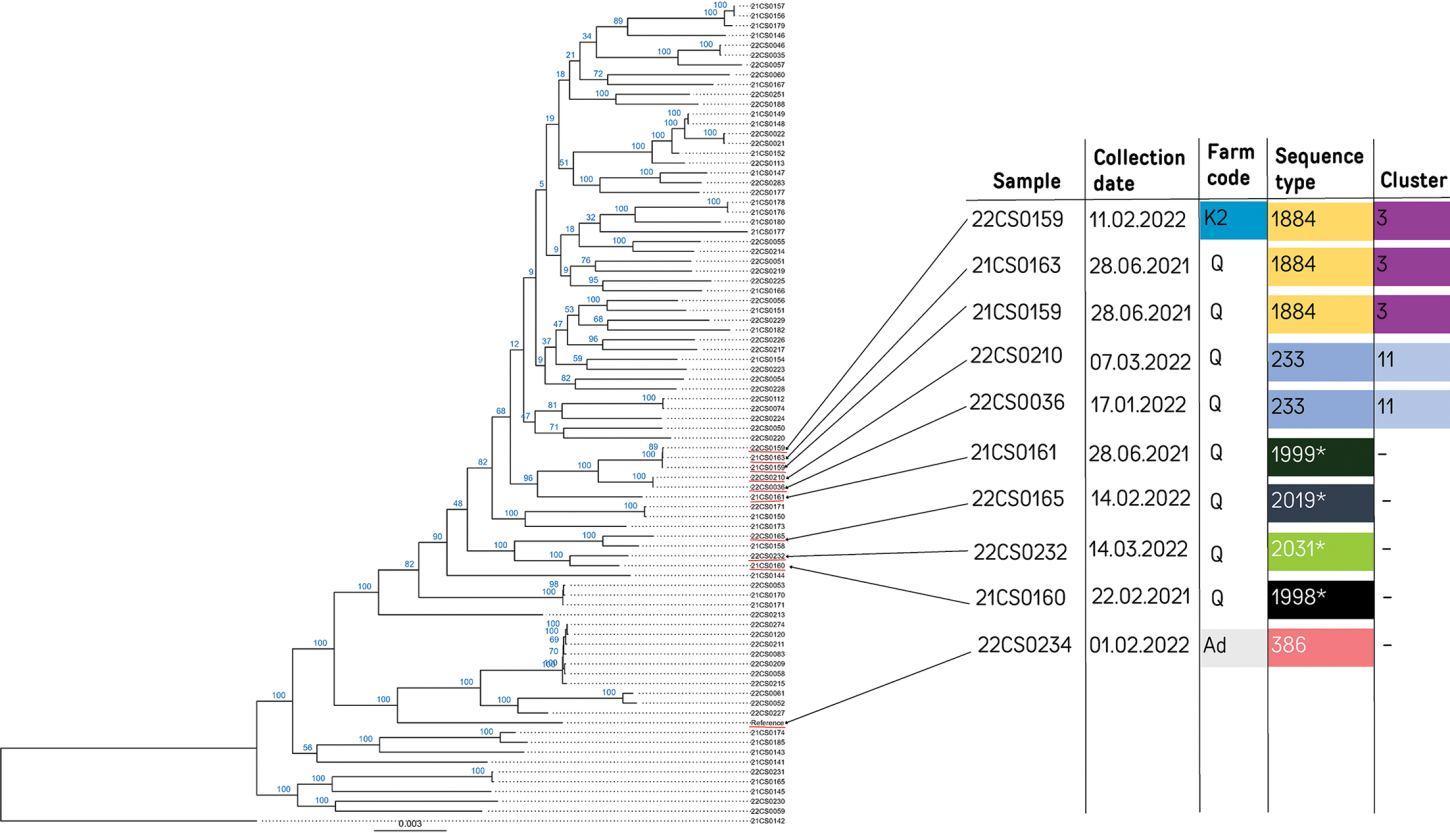
Phylogenetic tree (based on cgSNPs) of all 84 *S. uberis* strains. The plasmid was found only in the marked strains.

Out of ten strains harbouring p22CS0210, eight were isolated from farm Q, with the remaining two originating from farms K2 and Ad (Fig. 2). To compare the phylogeny of the chromosome (Fig. 4) to the phylogeny of the plasmid, reads of the ten strains were mapped to p22CS0210, and plasmid-SNP typing was performed (Supplementary material S7). While the majority of pairwise plasmid-SNP distances is zero, the largest pairwise difference within p22CS0210 was three cgSNPs. Several strains harbouring the specific plasmid differed in their chromosomes, while other strains carrying the plasmid shared high similarity in their chromosomes (Fig. 4). Eight strains from farm Q belonged to six STs (Fig. 4) and three chromosomal clusters (four strains are singletons), while their plasmid sequences differed in maximal one plasmid-SNP (six are completely identical). Moreover, the chromosome of strain 22CS0234 from farm Ad differed in more than 5000 cgSNPs to all other strains harbouring plasmid p22CS0210; however, its plasmid sequences differed in two to three cgSNPs from the other plasmids. On the other hand, three strains from cluster 3 (21CS0159, 21CS0163 and 22CS0159) collected from farm Q and one from farm K2 showed high similarities in their chromosomes (15–19 cgSNPs) and contained identical plasmid sequences. Additionally, two strains from cluster 11 (22CS0036 and 22CS0210) from farm Q displayed high genomic similarities (nine cgSNPs) in their chromosomes when being compared with each other.

## Discussion

*S. uberis* is a common environmental pathogen causing bovine mastitis [75]. To assess the AMR pattern and epidemiological situation of *S. uberis* in Thuringia, 84 isolates obtained from cattle with mastitis underwent AST against nine antibiotics, along with sequencing and genomic analysis. The phenotypic analysis was performed using a broth microdilution test according to CLSI guidelines.

### Phylogeography of *S. uberis* in Thuringia

*In silico* MLST was conducted to obtain an overview of the genomic diversity among the bacterial population. The diversity observed in Thuringia is in line with the findings from other geographic areas [76,78]. The abundance of newly identified STs may be attributed to the relatively modest size of the MLST database (around 2000 isolates in PubMLST as of November 2023). Additionally, the high diversity of strains within individual farms may indicate that mutations in the MLST genes can occur frequently, contributing to the overall diversity observed [79].

Considering higher-resolution genotyping through cgSNP analysis, the diversity among STs aligns with the diversity of cgSNPs indicated by an average pairwise distance of 4782 cgSNPs. This suggests the dissemination and coexistence of heterogeneous *S. uberis* strains rather than a contagious spread of clonal strains, as previously indicated [40, 79]. However, the persistence of genetically similar strains within one farm over a longer time period was observed, as well as the transmission of genetically highly similar strains between farms, which was also observed in other studies [24]. CgSNP-based clustering was conducted with an empirically determined threshold of 200 cgSNPs. The findings reveal no dominance of a particular strain in any specific region. Eight clusters exhibit a geographical preference, while two (clusters 1 and 2) are dispersed throughout Thuringia.

### Phenotypic and genotypic AMR patterns of *S. uberis* in Thuringia

Antibiotics were selected based on their relevance in veterinary use for cattle and human medicine [40,42,46], resulting in the testing of nine antibiotics. Three databases were used for a comprehensive scan of genetic markers for all substances included in those databases. A notable challenge in this analysis was the absence of breakpoints. CLSI has published clinical breakpoints for *S. uberis* in cattle only for three antibiotics (penicillin, ceftiofur and pirlimycin) [47]. For the remaining antibiotics, breakpoints were either adopted from the *Streptococcus viridans* group or from guidelines for human medicine as recommended by CLSI [47].

The high proportion of phenotypic resistance observed against tetracycline, approximately 42.9% for both doxycycline and tetracycline, corresponds to the findings in studies from Canada, Switzerland, Europe and Poland, in which resistance rates between 35 and 40% were reported [31,38, 80]. Studies from Portugal, Thailand, Brazil, Italy and the Czech Republic have reported higher resistance rates to tetracyclines, ranging from 50 to 85% [24,45, 81, 82]. In contrast, studies conducted in France and Switzerland revealed lower resistance prevalence with 18 and 28%, respectively [83,84]. In studies on *S. uberis*, genetic markers associated with tetracycline resistance are frequently detected [39,44, 45, 79, 80, 82, 85]. However, tetracyclines are not in the first line of treatment for bovine mastitis caused by *S. uberis* [86]. The strains 21CS0147, 21CS0150 and 22CS0219 were resistant to tetracycline, but no genetic marker conferring resistance was found, even though four databases were scanned. The detected phenotype might be due to a so far unknown genetic mechanism, which might be detected using a larger set of tetracycline-resistant strains without known markers in the future.

The absence of phenotypic beta-lactam resistance observed in this study is in line with the findings from Europe [80,82, 87], particularly studies from Bavaria and Hesse (Federal States neighbouring Thuringia) in which resistant strains were not identified [75,79]. In contrast, studies conducted outside of Europe present a different scenario. In Thailand, a 19% resistance rate to ceftiofur was reported, and in Brazil, 8% showed decreased susceptibility to ampicillin [24,85]. Additionally, in Canada, a small proportion of strains exhibited decreased susceptibility to ampicillin [31]. While penicillin susceptibility is phenotypically decreased for six strains in this study, resistance was not observed. This trend is consistent with other studies, such as those conducted in Canada [88,89], New Zealand [35] or several European countries [32,34], in which decreased susceptibility was observed. It is noteworthy to explore mutations found in genes coding for PBPs [35,79]. Compared to the reference strains, genetic differences in all 84 strains were detected in the genes coding for PBPs. While two unique substitutions were detected in genes coding for PBPs in two out of six strains with decreased susceptibility, these substitutions are not known to promote penicillin resistance in *S. uberis* [35,79]. No other mutation was found to be present in the six strains with decreased susceptibility, but not in the 78 susceptible strains. Still, many point mutations outside PBP-coding genes were detected among all strains. Previous studies reveal specific point mutations within PBP-coding genes linked to reduced susceptibility to beta-lactams in *Streptococci* and, in particular, *S. uberis* [34,35], which were not detected here. In *Streptococci*, mutations within and outside genes coding for PBPs conferred low levels of resistance, respectively, while the combination of mutations inside and outside PBP-coding genes cumulated in high-level resistance [90]. Hence, the accumulation of amino acid alterations within the PBP genes may represent an initial stage toward reduced susceptibility and eventual resistance to beta-lactams in *S. uberis* [84]. Our data will not allow further investigation along this line as many datasets need to be combined to find a statistically significant correlation between a genetic marker and penicillin susceptibility.

The absence of strains exhibiting resistance to vancomycin and the non-detection of known vancomycin resistance genes (*van* genes) is in accordance with the findings from studies in Portugal and Bangladesh [44,45].

Resistance to clindamycin was found in 22.6% of the strains tested in this study. This rate is lower than the one observed in the Czech Republic, with 30% resistant strains, but notably higher than the 1% resistance reported in Sweden [30,82]. Resistance to pirlimycin was found in 19% of the strains, a level similar to that reported in France and Canada [31,83]. However, higher rates of resistance were detected in Italy, Portugal and Brazil, ranging from 40 to 80% [24,45, 81]. Genes known to cause resistance to lincosamides are the *lnu* genes, which were detected in 100% of the strains with this phenotype. Pirlimycin is commonly used for treating bovine mastitis in Europe [45].

The erythromycin resistance rate observed in this study was 13%, which is comparable to the findings from Thailand, Canada and Switzerland [31,84, 85]. In France, the Netherlands and Italy, higher rates of around 20% were reported [83,87]. In Poland, only 6% of the isolated strains were found to be resistant [80]. All resistant strains in this study carried the gene *ermB* as also found in other studies [91].

In this study, we found that 39 of 84 strains (46%) showed phenotypic resistance to two or more antibiotics. A study from Italy reported that 57.7% of the tested strains were resistant to two or more classes of antimicrobial substances [81].

Overall, a strong correspondence between resistance phenotype and genotype was observed in this study. The discrepancy between genotypic and phenotypic resistance, as noted and discussed in the literature, is a known phenomenon [80,82]. Phenotypic resistance may be linked to specific point mutations rather than the presence of a particular resistance gene [80]. In this study, no specific point mutation correlating with decreased susceptibility to penicillin was identified. Additionally, the mere presence of a resistance gene does not guarantee its exclusive expression. Factors such as the distance of promoters to genes or their absence can affect gene expression, potentially leading to non-expression [80]. Another possible reason is the lack of data in the CLSI guidelines specifically tailored for veterinary strains, which may necessitate borrowing missing values from data on other animal species or norms established for human pathogens [92].

### Plasmids present in *S. uberis* in Thuringia

In this study, all strains resistant to erythromycin were also resistant to clindamycin and pirlimycin. These strains carried a specific plasmid, named p22CS0210*,* with two AMR genes, *ermB* and *lnuA*. The genes *ermC*, *ermB* and *ermR* have been recognized as primary determinants of macrolide–lincosamide–streptogramin resistance in streptococci and are believed to be horizontally transferred via plasmids between bacteria [37,93, 94]. The gene *lnuA* has been identified in many staphylococcal species, within plasmids playing a crucial role in the interspecies transfer of *lnuA* [95,96].

The plasmid p22CS0210 carrying genetic markers for resistance to macrolides and lincosamide was found in ten out of 84 *S. uberis* strains. The replication repressor *copG* identified on the plasmid is a typical replication repressor found in streptococci, for example on the plasmid pMV158 [97]. It is known to control plasmid copy number by repressing its own synthesis and that of the *repB* protein by binding to the *copG–repB* promoter region [97]. The mobilization gene located from position 2209 to 3708 includes a plasmid recombination protein that is also found in *S. suis*. This gene is associated with the mobilization of genetic material within the plasmid. The plasmid additionally carries the *ermB* gene, a known genetic marker conferring resistance to erythromycin. This gene is commonly found in streptococcal species and other bacteria such as *S. aureus* [98,99]. The other AMR gene, *lnu*A, confers lincosamide resistance and is often found on *S. aureus* plasmids [95,96].

The chromosomal phylogeny of strains carrying the plasmid p22CS0210 with the AMR genes *ermB* and *lnuA* is complex in comparison to the phylogeny of the plasmid sequences. While *S. uberis* chromosomes display high genetic diversity, sequences of the plasmid p22CS0210 are almost identical. The observation that genetically highly similar plasmids (zero to three cgSNPs among ten strains carrying p22CS0210) circulated within genetically highly different strains from different cgSNP clusters, STs, and farms points towards a potential horizontal gene transfer (HGT) of p22CS0210. If HGT is indeed the mechanism and the genetic markers present on the plasmid are expressed, it would spread cross-resistance to lincosamides and macrolides. While the plasmid sequences miss conjugation-related genes, direct HGT does not seem plausible. However, p22CS0210 could be transferred with the assistance of other conjugative elements [100]. In staphylococci, interspecies transfer of non-self-transmissible plasmids can be mobilized by conjugative plasmids, such as pIP501 [101], or by integrative and conjugative elements [102]. In *Streptococcus thermophilus*, plasmid transduction mediated by virulent phages has been shown to occur [103]. As we do not know where plasmid p22CS0210 originated, how frequent and how conserved it is among streptococcal species, its presence might also be due to multiple transfer events between different staphylococcal species [102]. Discrepancy was observed in one strain, 22CS0159, where *lnuA* and *ermB* were detected in Illumina sequencing data as well as the plasmid sequence. Still, the strain is not phenotypically resistant to the antibiotics pirlimycin, clindamycin and erythromycin, suggesting a potential loss of the plasmid during re-cultivation of this particular strain. The phenotypic testing was conducted after Illumina sequencing using a re-cultured colony of 22CS0159. Plasmid loss is a known phenomenon in bacterial populations and can occur under certain conditions or selective pressures [41].

## Conclusion

In conclusion, this study focused on the characterization of *S. uberis* strains isolated from cattle with mastitis in Thuringia and provides insights into the epidemiology, AMR patterns and genetic characteristics of *S. uberis* in Thuringia. Genotyping revealed genomic diversity among *S. uberis* strains in Thuringia with few possible contagious transmissions. The evaluation of AMR patterns involved phenotypic testing against nine antibiotics and genotypic analysis of resistance genes. Tetracycline resistance was prevalent, while beta-lactam resistance was absent. A specific plasmid p22CS0210 harbouring genetic markers for lincosamide and erythromycin resistance was identified in ten strains, and a hypothetical HGT event was suggested by distinct clustering in the phylogenetic tree. These findings highlight the importance of understanding AMR patterns in *S. uberis*, particularly in the context of mastitis treatment. The potential transmission of resistance via plasmids underscores the need for effective surveillance and management strategies to curb the spread of antibiotic-resistant *S. uberis* among individual animals and farms. Still, as was also shown for other mastitis-causing bacteria, milking hygiene, dry bedding material and high standards of farm management are essential [104].

## Supplementary material

10.1099/jmm.0.001887 Supplementary Material 1.

10.1099/jmm.0.001887 Supplementary Material 2.

10.1099/jmm.0.001887 Supplementary Material 3.

10.1099/jmm.0.001887 Supplementary Material 4.

10.1099/jmm.0.001887 Supplementary Material 5.

10.1099/jmm.0.001887 Supplementary Material 6.

10.1099/jmm.0.001887 Supplementary Material 7.

## Abbreviations

AMR: antimicrobial resistance
AST: antimicrobial susceptibility testing
CDS: coding sequences
cgSNP: core-genome single-nucleotide-polymorphism
CLSI: Clinical & Laboratory Standards Institute
ENA: European Nucleotide Archive
HGT: horizontal gene transfer
MEG: mobile genetic elements
MIC: minimum inhibitoy concentration
MLST: multilocus sequence typing
ONT: Oxford Nanopore Technologies
PBP: penicillin-binding protein
SCC: somatic cell count
ST: sequence type
TGD: tiergesundheitsdienste
WGS: whole-genome sequencing
